# Supplementary material for: Variability in porcine microRNA genes and its association with mRNA expression and lipid phenotypes
Source: Genet Sel Evol. 2021 May 4;53:43. doi: 10.1186/s12711-021-00632-3 (PMC8097994; doi:10.1186/s12711-021-00632-3)

Variability in porcine microRNA genes and its association with mRNA expression and lipid phenotypes

Emilio Mármol-Sánchez[1], María Gracia Luigi-Sierra[1], Anna Castelló[1,4], Dailu Guan[1], Raquel Quintanilla[2], Raul Tonda[3], Marcel Amills[1,4]

[1]Centre for Research in Agricultural Genomics (CRAG), CSIC-IRTA-UAB-UB, Universitat Autònoma de Barcelona, 08193 Bellaterra, Spain.

[2]Animal Breeding and Genetics Program, Institute for Research and Technology in Food and Agriculture (IRTA), Torre Marimon, 08140 Caldes de Montbui, Spain.

[3]CNAG-CRG, Centre for Genomic Regulation (CRG), Barcelona Institute of Science and Technology (BIST), Barcelona, Spain.

[4]Departament de Ciència Animal i dels Aliments, Universitat Autònoma de Barcelona, 08193 Bellaterra, Barcelona, Spain.

Corresponding autor: Marcel Amills. [1]Centre for Research in Agricultural Genomics (CRAG), CSIC-IRTA-UAB-UB, Universitat Autònoma de Barcelona, 08193 Bellaterra, Spain. Phone: +34 935636600. Email: Marcel.Amills@uab.es

Additional file 4: Fig. S1: Sequence homology between 12 porcine miRNAs harboring SNPs that have been genotyped in the Lipgen population (N = 345) and their corresponding human miRNA orthologous sequences.

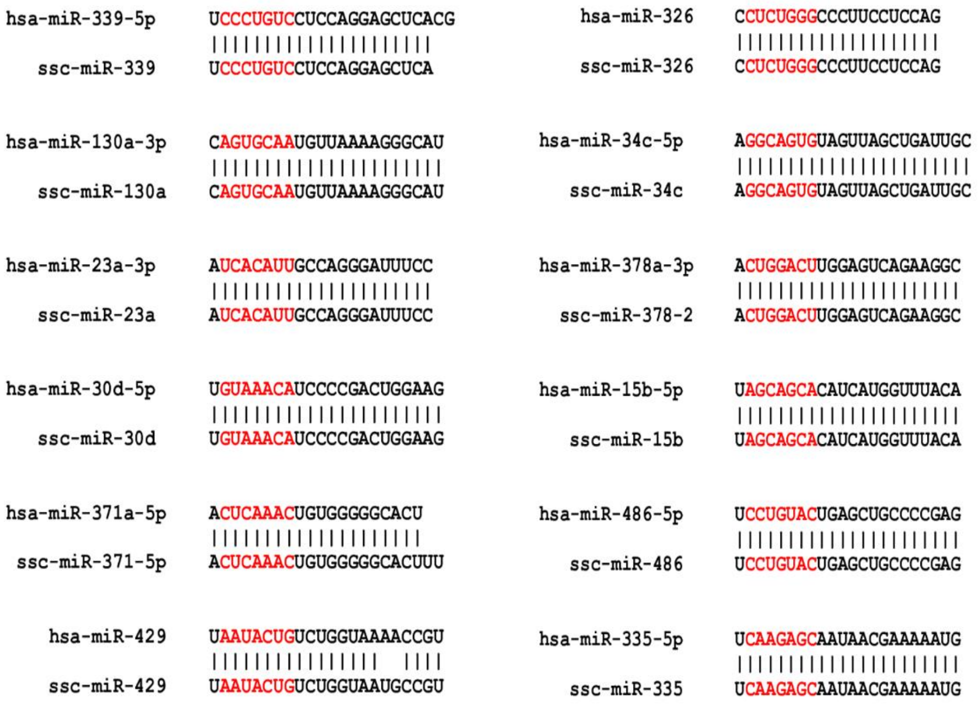

Additional file 8: Fig. S2: Number of SNPs present in each of the annotated porcine miRNA loci that display at least 1 SNP.

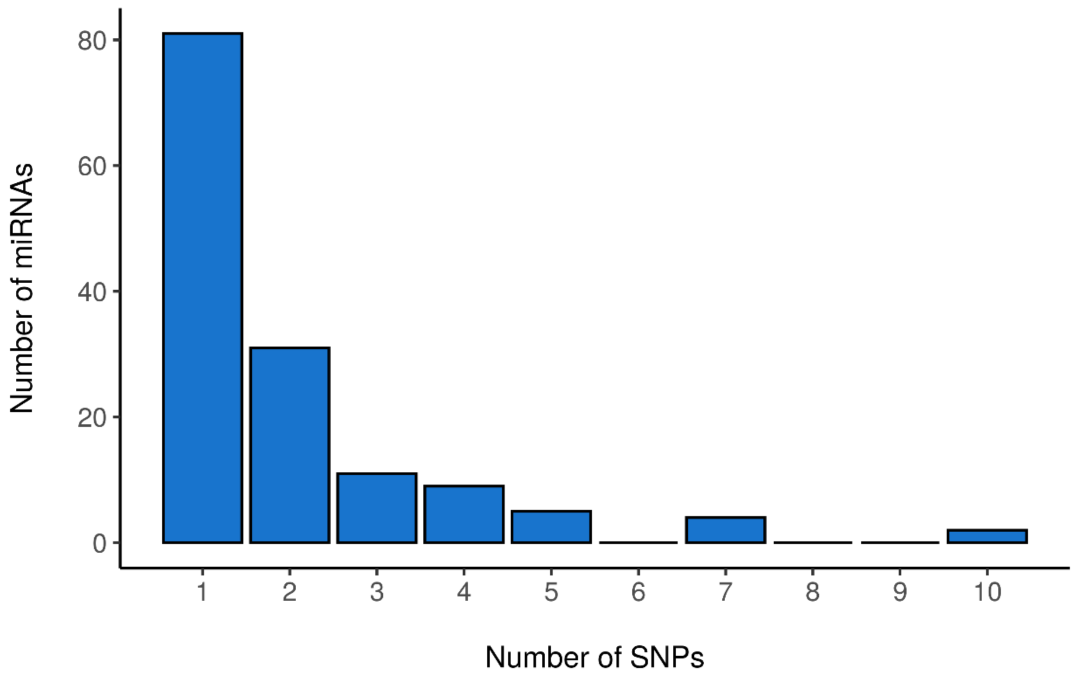

Additional file 9: Fig. S3: Venn Diagrams illustrating the sharing of SNPs located at the (**a**) *precursor*, (**b**) *mature* and (**c**) *seed* regions of miRNA loci among Asian domestic pigs (ADM), Asian wild boars (AWB), European domestic pigs (EDM) and European wild boars (EWB).

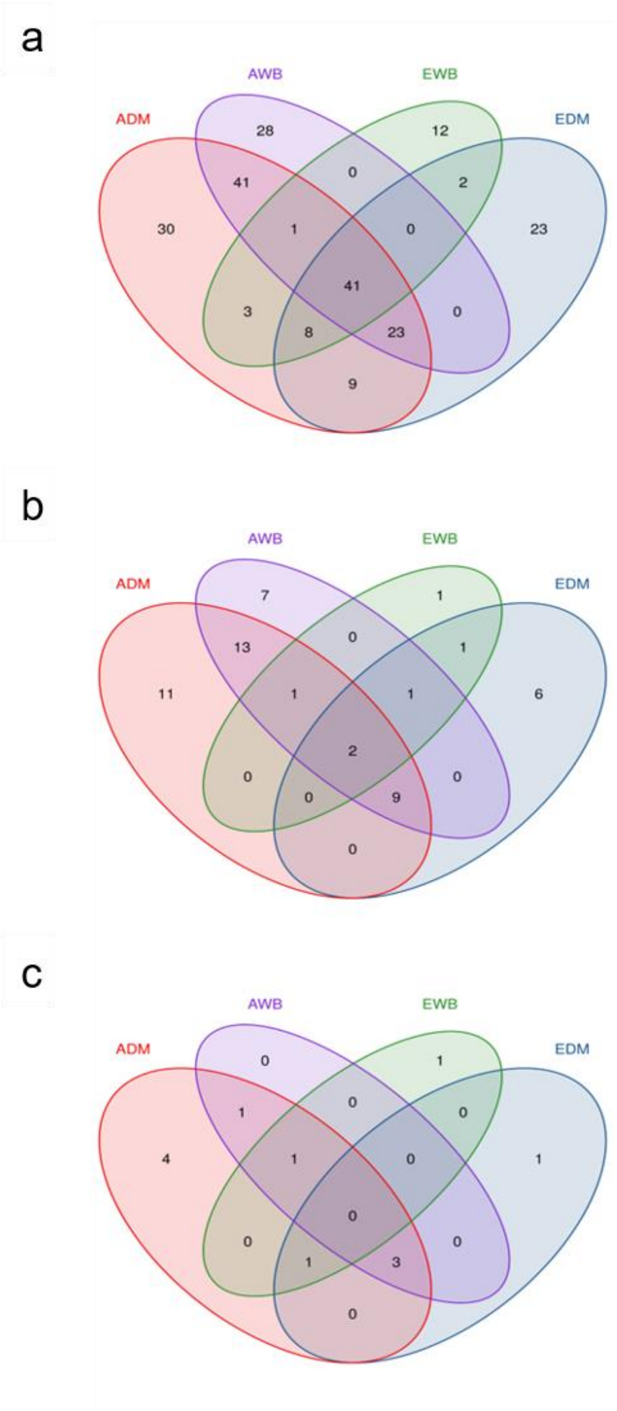

Additional file 12: Fig. S4: Boxplot distribution depicting whole-genome sequencing statistics for the five Duroc boars that sired the Lipgen population (N = 345).

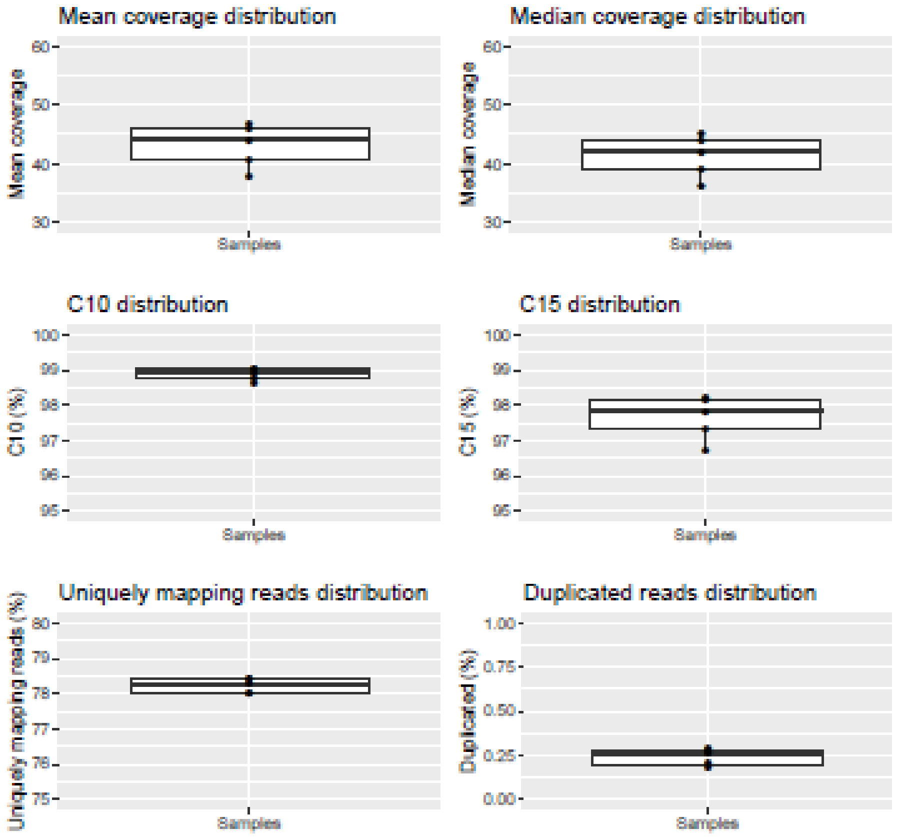

Supplement: Supplementary file 4 — Additional file 4: Figure S1. Sequence homology between 12 porcine miRNAs harboring SNPs that have been genotyped in the Lipgen population (N = 345) and their corresponding human miRNA orthologous sequences. Figure S2. Number of SNPs present in each of the annotated porcine miRNA loci that display at least one SNP. Figure S3. Venn Diagrams illustrating the sharing of SNPs located at the (a) precursor, (b) mature and (c) seed regions of miRNA loci among Asian domestic pigs (ADM), Asian wild boars (AWB), European domestic pigs (EDM) and European wild boars (EWB). Figure S4. Boxplot distribution depicting whole-genome sequencing statistics for the five Duroc boars that sired the Lipgen population (N = 345). [file 12711_2021_632_MOESM4_ESM.pdf]
